# Low serum calcium is associated with poor renal outcomes in chronic kidney disease stages 3–4 patients

**DOI:** 10.1186/1471-2369-15-183

**Published:** 2014-11-21

**Authors:** Lee-Moay Lim, Hung-Tien Kuo, Mei-Chuan Kuo, Yi-Wen Chiu, Jia-Jung Lee, Shang-Jyh Hwang, Jer-Chia Tsai, Chi-Chih Hung, Hung-Chun Chen

**Affiliations:** Division of Nephrology, Department of Internal Medicine, Kaohsiung Medical University Hospital, Kaohsiung Medical University, No. 100, Tzyou 1st Road, Kaohsiung, 807 Taiwan; Department of Internal Medicine, Pingtung Hospital, Ministry of Health and Welfare, No. 270 Zihyou Road, Pingtung City, Pingtung County Taiwan; Faculty of Renal Care, College of Medicine, Kaohsiung Medical University, No. 100, Shih-Chuan 1st Road, Kaohsiung, 807 Taiwan

**Keywords:** Calcium, Chronic kidney disease, Renal replacement therapy

## Abstract

**Background:**

Mineral disorders are associated with adverse renal outcomes in chronic kidney disease (CKD) patients. Previous studies have associated hypercalcemia and hypocalcemia with mortality; however, the association between serum calcium and renal outcome is not well-described. Whether adding calcium besides phosphorus or in the form of calcium-phosphorus (Ca × P) product into the model of survival analysis could improve the prediction of renal outcomes is not known.

**Methods:**

A prospective cohort of 2144 outpatients with CKD stages 3–4 was evaluated. Cox proportional hazard analysis was performed according to calcium quartiles.

**Results:**

The mean calcium level was 9.2 ± 0.7 mg/dL. Low serum calcium (<9.0 mg/dL) was associated with increased risk of requiring renal replacement therapy (RRT) (hazards ratio [HR]:2.12 (95% CI: 1.49–3.02, P <0.05) and rapid renal function progression (odds ratio [OR]: 1.65 (95% CI: 1.19–2.27, P <0.05) compared with high serum calcium (>9.8 mg/dL). Adding calcium into the survival model increased the integrated discrimination improvement by 0.80% (0.12% – 1.91%) while calcium-phosphorus product did not improve risk prediction.

The combination of high serum phosphorus (>4.2 mg/dL) and low serum calcium (<9.1 mg/dL) was associated with the highest risk of RRT (HR:2.31 (95% CI: 1.45–3.67, P < 0.05).

**Conclusion:**

Low serum calcium is associated with increased risk of RRT and rapid renal function progression in CKD stage 3–4 patients. The integration of serum calcium and phosphorus, but not calcium-phosphorus product should be considered in a predictive model of renal outcome.

**Electronic supplementary material:**

The online version of this article (doi:10.1186/1471-2369-15-183) contains supplementary material, which is available to authorized users.

## Background

Bone mineral disorders have been implicated as a risk factor for mortality and renal replacement therapy (RRT) in chronic kidney disease (CKD) patients [1–3]. Several observational studies have shown a significant association between high phosphorus levels with the progression of CKD [4, 5] and RRT [4, 6]. Nevertheless, recent studies have discovered the relationship between low plasma 25-hydroxyvitamin D (25[OH]D) and 1,25-dihydroxyvitamin D (1,25[OH]_2_D) with renal disease progression [7], the initiation of dialysis [8], and death [7, 8]. Although the exact mechanisms of renal damage are not completely understood, putative mechanisms could be vascular calcification and arteriosclerosis induced by high serum phosphorus [9], increased calcium and calcium-phosphorus (Ca × P) product [10].

The potential role of serum calcium in the prediction of CKD progression is not well-established. In previous studies, Young et al. identified that hypercalcemia plays a critical modifying role in the pathogenesis of ischemic and toxic renal injury [11], and the association between hypercalcemia and acute kidney injury was observed in malignancy, hyperparathyroidism, and sarcoidosis [12]. A study by Schwarz et al. suggested that lower serum calcium was associated with higher risk of CKD progression; however, this association was not statistically significant [2]. There is no study directly aimed to evaluate the role of calcium as a risk factor for poor renal outcome. Moreover, previous case reports have documented hypocalcemia associated congestive heart failure in both pediatric and adult population because myocardial contractility may decline in acute and chronic hypocalcemia [13, 14]. The researches regarding relationship between serum calcium and cardiovascular system in CKD are limited. Therefore, in this study, we hypothesized that serum calcium is an independent prognostic marker of rapid renal function progression, the requirement for RRT and deterioration in cardiac performance in an observational cohort of CKD stages 3–4 patients.

## Methods

### Study population and data collection

A prospective cohort of CKD patients from integrated CKD care programs in Kaohsiung City was evaluated. CKD was defined and staged according to the definition of the Kidney Disease Outcomes Quality Initiative (KDOQI), and classified using the patients’ baseline estimated glomerular filtration rate (eGFR) [15]. The patients (n = 3749) were recruited from the nephrology outpatient departments of 2 hospitals, and were treated under integrated CKD care programs from November 11, 2002 until May 31, 2009, and followed up until July 31, 2010. The inclusion criteria were being a CKD patient and not receiving RRT. The exclusion criteria were acute kidney injury, defined as more than a 50% reduction in the eGFR within 3 months, patients who were lost to follow-up in less than 3 months (n = 90),CKD stage 1, 2 patients due to small sample size (n = 356) and CKD stage 5 patients. The final study population consisted of 2144 patients with CKD stages 3–4.

At the baseline visit, socio-demographic characteristics, medical history, lifestyle behaviors, and current medications were recorded. The patients’ medical histories were confirmed by doctors’ chart-reviews. The presence of diabetes mellitus (DM) and hypertension was defined according to clinical diagnosis. Cardiovascular disease (CVD) was defined as a clinical diagnosis of heart failure, acute or chronic ischemic heart disease, or cerebrovascular accident. The patients’ biochemistry measurements were collected on baseline visit, and then every 3 months. The laboratory data from 3 months prior to the baseline visit to 3 months after the baseline visit were averaged and analyzed. The normal range of serum phosphate and calcium in our institution are 2.5-4.6 mg/dl and 8.4-10.2 mg/dl respectively. The total calcium was measured and corrected for serum albumin concentration using the following formula: corrected calcium (mmol/L) = total calcium (mmol/L) +0.02*(40 g/L - serum albumin in grams per liter) [16]. Corrected total calcium level is analyzed in our models. Patients’ calcium levels were divided into quartiles Ca Q_1_–Q_4_ according to cut-off values at 9.0 mg/dL, 9.4 mg/dL, and 9.8 mg/dL.

### Echocardiographic assessment

All patients received echocardiography examination annually when they entered CKD stage 5. The echocardiographic examination was performed by experienced cardiologists who were blind to clinical data using transthoracic echocardiography (Vivid 7, General Electric Medical Systems, Horten, Norway). Left ventricular mass index (LVMI) was calculated by dividing left ventricular mass by body surface area [17].

### Ethics statement

The study protocol was approved by the Institutional Review Board of the Kaohsiung Medical University Hospital (KMUH-IRB-990198). Informed consents were obtained in written form and all clinical investigation was conducted according to the principles expressed in the Declaration of Helsinki. The patients gave consent for the publication of the clinical details.

### Renal outcome

RRT was defined as hemodialysis, peritoneal dialysis, or renal transplantation, or as RRT for more than 3 months. The timing of administration of RRT was according to the regulations of the Bureau of the National Health Insurance of Taiwan for laboratory data, nutritional status, uremic status, and serum creatinine (>6 mg/dL). Patients’ charts were reviewed and matched with the database of the Taiwan Society of Nephrology to identify the cases receiving RRT.

A rapid renal function progression was defined as an eGFR slope < -5 mL/min/1.73 m^2^/y. Renal function was quantified using the eGFR derived from the simplified Modification of Diet in Renal Disease (MDRD) study equation. The average eGFR slope (mL/min/1.73 m^2^/y) for each patient was calculated using linear regression with varying intercepts and varying slopes, but without covariates, for the estimation of annual changes in eGFR. Other cut-off values were also applied in sensitivity tests.

To compare the performances of a new predictive model versus an old predictive model, the integrated discrimination improvement (IDI) was applied, which calculates integrated sensitivity (IS) and integrated one minus specificity (IP) in the old and new models as IDI = (IS_new_ - IS_old_) - (IP_new_ - IP_old_) [18].

### Statistical analysis

The baseline characteristics of all patients are expressed as percentages for categorical data, in mean ± standard deviation (SD) for continuous variables with approximately normal distribution, and median and interquartile ranges for continuous variables with skewed distribution.

Cox proportional hazards analyses were used to evaluate the hazard ratios (HR) for RRT associated with calcium levels. Multivariate logistic regression analyses were used to evaluate the association between calcium and rapid renal disease progression. Covariates were included into these models if the *P* value was <0.05 in univariate analysis and skewed distributed continuous variables were log-transformed to attain normal distribution. The adjusted covariates included age, sex, eGFR, urine protein to creatinine (UPCR) ratio log, CVD, DM, glycosylated hemoglobin, mean blood pressure (MBP), hemoglobin, albumin, C-reactive protein, body mass index (BMI), cholesterol log, phosphorus, use of phosphate binders, and iPTH. A *P* value <0.05 was considered statistically significant. Statistical analysis was performed using the R 2.15.2 software (R Foundation for Statistical Computing, Vienna, Austria) and the Statistical Package for Social Sciences version 18.0 for Windows (SPSS Inc, Chicago, IL, USA).

## Results

### Baseline characteristics of the study group according to calcium quartiles

Table 1 displays the baseline characteristics of the study patients. The mean calcium level was 9.2 ± 0.7 mg/dL. The mean eGFR was 33.2 ± 11.9 mL/min/1.73 m^2^. The patients from the Ca Q_1_ subgroup were more likely to have lower MBP, lower eGFR, hemoglobin, and bicarbonate levels, and higher UPCR and iPTH levels than were the other calcium quartile groups. The use of phosphate binders showed non-significant differences between the groups.

**Table 1:** **Baseline characteristic of CKD stages 3–4 patients according to quartiles of serum calcium level**

		Quartile of serum calcium level (mg/dL)				
		Q _1_	Q _2_	Q _3_	Q _4_	
	All patients	(<9.0)	(9.0 - 9.4)	(9.4 - 9.8)	(>9.8)	***p***for trend
No. of patients	2144	544	452	588	560	-
***Demographic data***						
Age, yrs	64.2 ± 13.5	64.1 ± 14.3	64.8 ± 13.7	63.8 ± 13.2	64.1 ± 13.0	0.636
Gender (female)	756 (35.3%)	179 (32.9%)	149 (33.0%)	207 (35.2%)	221 (39.5%)	0.017
Diabetes mellitus	940 (43.8%)	209 (38.4%)	184 (40.7%)	265 (45.1%)	282 (50.4%)	<0.001
Cardiovascular disease	381 (17.8%)	101 (18.6%)	92 (20.4%)	94 (16.0%)	94 (16.8%)	0.197
Hypertension	1360 (63.4%)	386 (71.0%)	303 (67.0%)	364 (61.9%)	307 (54.8%)	<0.001
Smoker (recent)	279 (13.0%)	70 (12.9%)	56 (12.4%)	78 (13.3%)	75 (13.4%)	0.711
Mean BP, mmHg	99.4 ± 13.5	97.9 ± 13.1	99.1 ± 12.9	100.5 ± 14.8	100.1 ± 12.7	0.002
Body mass index, kg/m^2^	25.0 ± 3.9	24.6 ± 3.8	25.2 ± 3.8	25.1 ± 4.1	25.3 ± 4.1	0.017
***Laboratory results***						
eGFR, ml/min/1.73 m^2^	33.2 ± 11.9	31.2 ± 11.8	33.4 ± 11.8	34.5 ± 11.8	33.5 ± 11.9	0.001
Hemoglobin, g/dL	11.9 ± 2.1	11.5 ± 2.0	12.1 ± 2.1	12.1 ± 2.1	12.0 ± 2.1	<0.001
Albumin, g/dL	3.9 ± 0.5	3.9 ± 0.4	3.9 ± 0.4	3.9 ± 0.5	3.8 ± 0.6	0.003
GPT, U/I	26.3 ± 25.1	24.4 ± 23.8	25.5 ± 24.6	27.1 ± 27.4	28.2 ± 24.0	0.006
Cholesterol, mg/dL	192.0 (165.0-223.5)	187.0 (160.0-213.0)	190.0 (163.0-217.8)	194.0 (168.8-224.0)	198.0 (167.6-232.8)	<0.001
Triglyceride, mg/dL	127.0 (93.0-189.0)	117.0 (86.3-171.7)	123.0 (89.0-172.8)	134.0 (97.0-188.0)	141.0 (100.0-212.8)	<0.001
WBC, x10^3^/uL	7.2 ± 2.3	7.1 ± 2.4	7.1 ± 2.1	7.1 ± 2.1	7.5 ± 2.4	0.012
CRP, mg/dL;	1.0 (0.3-4.6)	1.1 (0.5-4.0)	1.0 (0.4-3.3)	0.9 (0.3-4.2)	1.2 (0.2-7.3)	0.993
HbA1c, mg/dL;	6.6 ± 1.7	6.4 ± 1.5	6.3 ± 1.4	6.7 ± 1.7	7.0 ± 1.8	<0.001
UPCR, mg/g	702.8 (263.0-1697.7)	800.7 (334.6-1778.8)	721.3 (269.9-1670.1)	615.6 (229.1-1698.2)	659.8 (244.9 - 1698.6)	0.016
Sodium, mEq/L	138.6 ± 3.4	138.2 ± 3.4	138.7 ± 3.1	138.9 ± 3.4	138.8 ± 3.6	0.003
Potassium, mEq/L	4.3 ± 0.5	4.3 ± 0.5	4.2 ± 0.5	4.3 ± 0.5	4.4 ± 0.5	<0.001
Phosphorus, mg/dL	3.9 ± 0.8	4.0 ± 0.9	3.9 ± 0.8	3.9 ± 0.8	3.9 ± 0.7	0.019
Calcium, mg/dL	9.2 ± 0.7	8.6 ± 0.5	9.1 ± 0.3	9.3 ± 0.3	10.0 ± 0.5	<0.001
Ca x Pi	36.2 ± 7.5	34.2 ± 7.4	35.1 ± 7.0	36.6 ± 7.5	38.5 ± 7.2	<0.001
PTH, pg/mL	68.4 ± 79.5	96.2 ± 105.8	66.3 ± 68.6	61.6 ± 72.2	50.4 ± 54.4	<0.001
Bicarbonate, mEq/L	23.4 ± 3.6	22.6 ± 3.8	23.6 ± 3.5	23.8 ± 3.5	23.8 ± 3.7	<0.001
Uric acid, mg/dL	7.8 ± 1.9	7.8 ± 1.9	7.7 ± 1.9	7.7 ± 1.8	7.8 ± 2.0	0.588
Phosphate binder	381 (17.8%)	105 (19.3%)	86 (19.0%)	92 (15.6%)	98 (17.5%)	0.129
***Outcome***						
Follow-up, days	1085(682–1673)	1061 (673–1608)	1059 (676–1575)	1092 (688–1686)	1191 (705–1749)	0.017
LVMI	118.5 (45.0)	129.2 (42.9)	121.9 (56.3)	112.3 (42.7)	114.1 (37.6)	0.037
eGFR slope, ml/min/1.73 m^2^/yr	-1.9 (-5.4-0.5)	-2.6 (-6.2-0.1)	-1.7 (-4.7-0.5)	-1.9 (-5.6-0.5)	-1.4 (-4.9-1.1)	<0.001
Rapid renal progression	574 (27.0%)	172 (31.9%)	115 (25.4%)	151 (25.7%)	136 (24.5%)	0.029
RRT	294 (13.7%)	107 (19.7%)	54 (11.9%)	73 (12.4%)	60 (10.7%)	<0.001
Death	270 (12.6%)	56 (10.3%)	53 (11.7%)	65 (11.1%)	96 (17.1%)	<0.001

*CKD*, Chronic kidney disease; *BP*, Blood pressure; *eGFR*, Estimated glomerular filtration rate; *GPT*, Glutamic pyruvic transaminase; *WBC*, White blood cell count; *CRP*, C-reactive protein; *HbA1c*, Glycosylated hemoglobin; *UPCR*, Urine protein to creatinine ratio; *Ca x Pi*, Calcium phosphate product; *PTH*, Parathyroid hormone; *RRT*, Renal Replacement Therapy; *LVMI*, Left ventricular mass index; *LVH*, Left ventricular hypertrophy.

Continuous variables are expressed as mean ± standard deviation or median (interquartile range), and categorical variables are expressed as number (percentage). *P* for trend <0.05 indicates a significant trend for increasing calcium levels.

Table 2 shows the variables associated with serum calcium in CKD stages 3–4. Female sex, DM, hemoglobin, HbA1c and cholesterol log levels showed positive correlations with serum calcium. In contrast, CVD, eGFR, albumin, phosphorus, iPTH, and UPCR log levels showed negative correlations with serum calcium.

**Table 2:** **Multivariate linear regression for serum calcium in CKD stage 3–4 patients**

Variables	β coefficient	95% CI	***p***-value
Age, yrs	-0.001	-0.003 to 0.001	0.370
Gender (male vs. female)	0.143	0.084 to 0.202	<0.001
Diabetes mellitus	0.088	0.030 to 0.147	0.003
Cardiovascular disease	-0.102	-0.163 to -0.041	0.001
Mean BP, mmHg	0.001	-0.001 to 0.003	0.145
Body mass index, kg/m^2^	0.002	-0.005 to 0.009	0.562
eGFR, ml/min/1.73 m^2^	-0.004	-0.006 to -0.001	0.010
Hemoglobin, g/dL	0.025	0.009 to 0.041	0.003
Albumin, g/dL	-0.211	-0.270 to -0.151	<0.001
Log (Cholesterol)	0.467	0.222 to 0.711	<0.001
Ln (CRP)	0.010	-0.020 to 0.040	0.504
HbA1c,mg/dL;	0.036	0.019 to 0.054	<0.001
Log (UPCR)	-0.110	-0.168 to -0.052	<0.001
Phosphorus, mg/dL	-0.062	-0.097 to -0.027	<0.001
PTH, pg/mL	-0.002	-0.002 to -0.002	<0.001
Phosphate binder	-0.031	-0.105 to 0.044	0.419

*CKD*, Chronic kidney disease; *BP*, Blood pressure; *eGFR*, Estimated glomerular filtration rate; *CRP*, C-reactive protein; *HbA1c*, Glycosylated hemoglobin; *UPCR*, Urine protein to creatinine ratio; *PTH*, parathyroid hormone.

*P* <0.05 indicates a significant associated with serum calcium levels.

### Calcium and left ventricular hypertrophy

Table 1 shows the echocardiography findings according to quartiles of serum calcium. Patients with serum calcium <9.0 mg/dL have higher LVMI compared with others quartiles, which were 129.2 g/m^2^ (P < 0.05) and 42.9% respectively. Lower serum calcium demonstrated an increased risk of LVH with an adjusted OR of 1.64 (95% CI = 0.98–2.95, *P* < 0.05) (Additional file 1: Table S1).

### Calcium and RRT

**Figure 1:**
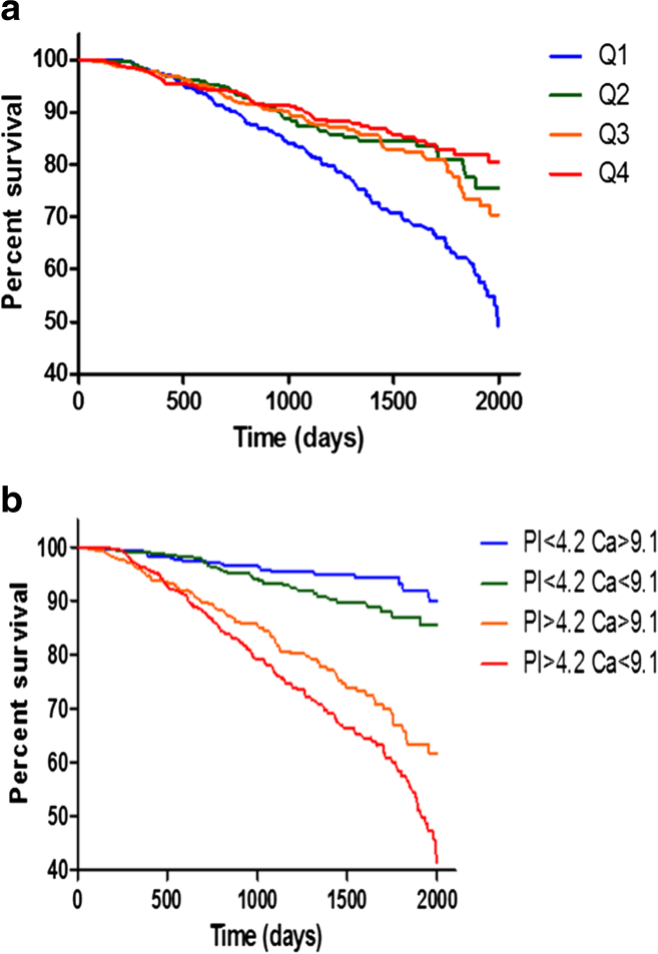
**Renal survival curves according to calcium and phosphorus level. a** Risk for renal replacement therapy (RRT) according to serum calcium. **b** Mineral disorder and risk for renal replacement therapy (RRT).

**Figure 2:**
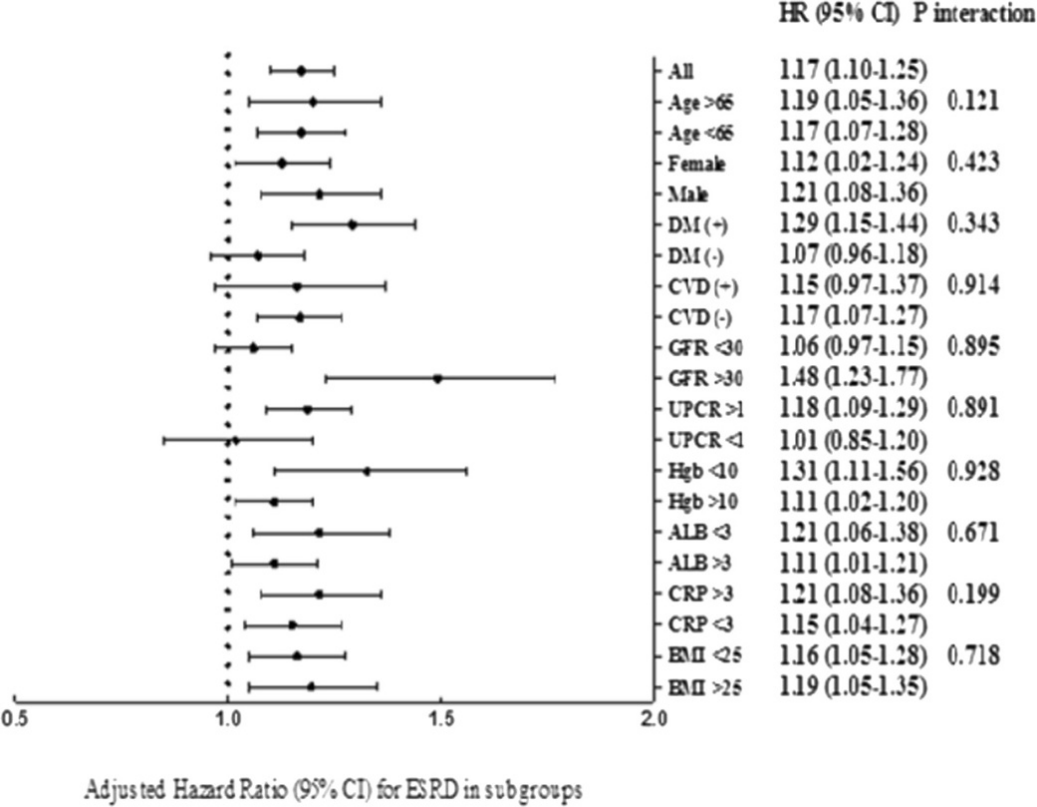
**Adjusted hazard ratio of renal replacement therapy (RRT) for 1 mg/dL decreased of serum calcium in different subgroup.**

During a median 2.9-year follow-up period, there were total of 294 patients (13.7%) commencing RRT (Table 1). The percentage of patients requiring RRT was highest in the Ca Q_1_ group (19.7%) compared other groups. Lower serum calcium was associated with increased risk of requiring RRT, with an adjusted HR of 2.12 (95% CI = 1.49–3.02, *P* <0.05) when comparing the Ca Q_1_ and Ca Q_4_ groups (Table 3). Figure 1a shows the renal survival curves for the calcium quartile groups. We also performed a survival analysis for RRT in the pre-specified subgroups. Non-significant interactions were observed (Figure 2).

**Table 3:** **Association of calcium with renal replacement therapy and rapid renal function progression**

	Quartile of serum calcium level (mg/dL)			
	Q _1_	Q _2_	Q _3_	Q _4_
HR (95% CI)	(<9.0)	(9.0 - 9.4)	(9.4 - 9.8)	(>9.8)
***Renal replacement therapy***				
Unadjusted	2.12 (1.55-2.91)*	1.26 (0.90-1.76)	1.15 (0.79-1.68)	1 (reference)
Model 1	1.52 (1.10-2.10)*	1.32 (0.94-1.86)	0.97 (0.67-1.42)	1 (reference)
Model 2	1.64 (1.19-2.26)*	1.32 (0.94-1.86)	1.05 (0.72-1.54)	1 (reference)
Model 3	2.34 (1.65-3.31)*	1.58 (1.11-2.25)*	1.51 (1.01-2.25)*	1 (reference)
Model 4	2.12 (1.49-3.02)*	1.50 (1.05-2.15)*	1.42 (0.95-2.12)	1 (reference)
***Rapid renal function progression***				
Unadjusted	1.44 (1.11-1.88)*	1.18 (0.90-1.54)	0.94 (0.70-1.26)	1 (reference)
Model 1	1.42 (1.06-1.91)*	1.28 (0.95-1.71)	0.93 (0.67-1.28)	1 (reference)
Model 2	1.56 (1.15-2.10)*	1.30 (0.97-1.75)	0.98 (0.71-1.36)	1 (reference)
Model 3	1.72 (1.25-2.35)*	1.40 (1.03-1.91)*	1.15 (0.82-1.61)	1 (reference)
Model 4	1.65 (1.19-2.27)*	1.35 (0.99-1.84)	1.11 (0.79-1.56)	1 (reference)

Model 1 adjusts for age, gender, eGFR, log(UPCR).

Model 2 adjusts for covariates in model 1 plus diabetes mellitus, cardio vascular disease, HbA1c, mean BP.

Model 3 adjusts for covariates in model 2 plus hemoglobin, albumin, log(cholesterol), ln(CRP), body mass index.

Model 4 adjusts for covariates in model 3 plus phosphorus, phosphate binder, PTH.

*HR*, Hazard ratio; *eGFR*, Estimated glomerular filtration rate; *UPCR*, Urine protein to creatinine ratio; *HbA1c*, Glycosylated hemoglobin; *BP*, Blood pressure; *PTH*, Parathyroid hormone.

*(*p* <0.05) indicates a significantly different from reference group.

### Calcium and rapid renal function progression

The median eGFR slope was -1.9 mL/min/1.73 m^2^/y. The Ca Q_1_ group was associated with the greatest renal functional decline, with an eGFR slope of -2.6 mL/min/1.73 m^2^/y compared with -1.4 mL/min/1.73 m^2^/y in the Ca Q_4_ group. Table 3 also lists the odds ratios for rapid renal function progression, defined as an eGFR slope < -5 mL/min/1.73 m^2^/y. Lower serum calcium was associated with increased risk of rapid renal function progression, with an adjusted OR of 1.65 (95% CI = 1.19–2.27) when comparing the Ca Q_1_ and Ca Q_4_ groups (Table 3). However, higher serum calcium showed no association with increased risk of rapid renal function progression when comparing the Ca Q_4_ and Ca Q_3_ groups.

### Serum calcium and phosphorus are predictive of requiring RRT

To evaluate the combined effects of serum calcium and phosphorus on the requirement for RRT, we further divided the patients into calcium (≥9.1 mg/dL and <9.1 mg/dL) and phosphorus (≥4.2 mg/dL and <4.2 mg/dL) subgroups. The group with lower serum calcium (<9.1 mg/dL) and higher phosphorus (>4.2 mg/dL) was associated with increased risk of requiring RRT, with an adjusted HR of 2.31 (95% CI = 1.45–3.67, *P* <0.05) (Table 4). Figure 1b shows the renal survival curves according to calcium and phosphorus levels. When serum calcium was added into the survival model, the IDI increased, with a discrimination slope 0.08% higher than the original (Model 3 vs Model 1, IDI 0.80% (0.12% – 1.91%); Table 5). Conversely, Ca × P was add into the survival model, the discrimination slope was 0.05% lower than the original (Model 4 vs Model 1, IDI -0.50% (-0.94% –0.07%); Table 5).

**Table 4:** **Mineral disorder and risk of renal outcomes**

		Hazard ratio (95% CI) for renal replacement therapy		Odd ratio (95% CI) for rapid renal function progression	
P > 4.2 mg/dl	Ca > 9.1 mg/dl	Unadjusted	Adjusted	Unadjusted	Adjusted
No	Yes	1 (reference)	1 (reference)	1 (reference)	1 (reference)
No	No	1.58 (0.94-2.66)	1.21 (0.71-2.07)	1.15 (0.85-1.57)	1.07 (0.76-1.51)
Yes	Yes	4.76 (3.01-7.53)*	1.53 (0.94-2.47)	2.37 (1.76-3.20)*	1.26 (0.88-1.79)
Yes	No	6.64 (4.28-10.32)*	2.31 (1.45-3.67)*	2.70 (2.02-3.59)*	1.68 (1.20-2.35)*

Adjusts the variables including age, gender, eGFR, log(UPCR), diabetes mellitus, cardio vascular disease, HbA1c, mean BP, hemoglobin, albumin, log(cholesterol), ln(CRP), body mass index, phosphate binder and PTH.

*eGFR*, Estimated glomerular filtration rate; *UPCR*, Urine protein to creatinine ratio; *HbA1c*, Glycosylated hemoglobin; *BP*, Blood pressure; *PTH*, Parathyroid hormone.

*(*p* <0.05) indicates a significantly different from reference group.

**Table 5:** **Integrated discrimination improvement (IDI) of adding phosphorus, calcium or calcium x phosphorus in the survival model**

	IDI % (95% CI)	
	vs. Model 0	vs. Model 1
Model 0	-	-
Model 1 (Model 0 + Pi)	0.74 (0.06 – 1.53)*	-
Model 2 (Model 0 + Ca)	0.82 (0.00 – 1.98)*	0.07 (-0.97 – 2.46)
Model 3 (Model 0 + Pi + Ca)	1.55 (0.16 – 2.90)*	0.80 (0.12 – 1.91)*
Model 4 (Model 0 + Ca x Pi)	0.23 (-0.16 – 0.80)	-0.50 (-0.94 – 0.07)

Model 0 included the following variables: age, gender, eGFR, log(UPCR), diabetes mellitus, cardio vascular disease, HbA1c, mean BP, hemoglobin, albumin, log(cholesterol), ln(CRP), body mass index, phosphate binder and PTH.

*eGFR*, Estimated glomerular filtration rate; *UPCR*, Urine protein to creatinine ratio; *HbA1c*, Glycosylated hemoglobin; *BP*, Blood pressure; *PTH*, Parathyroid hormone.

*P < 0.05.

## Discussion

In this study, we showed that low serum calcium (<9.0 mg/dL) is an independent prognostic marker of requiring RRT and rapid renal function progression in CKD stages 3–4 patients. We did not observe any association between high serum calcium (>9.8 mg/dL) and renal outcome.

Previous epidemiological studies on CKD patients have focused on the association between bone mineral disorder and outcomes [4, 7, 19, 20]. Although these studies have extensively investigated several of the abnormalities that characterize CKD mineral and bone disorder (CKD-MBD), such as serum phosphorus, iPTH, vitamin D, and FGF-23, and their roles as biomarkers for prediction of outcomes in a CKD population, few studies have evaluated serum calcium and renal outcome in CKD. Recently, Lundberg et al. identified that reduced serum calcium (per 0.1 mmol/L) was associated with higher time-averaged albuminuria, and predictive of deterioration in renal function, in an observational cohort of IgA nephropathy patients [21]. Our study results suggested that serum calcium should be included in models for the estimation of the risk of renal events. Serum calcium is a commonly collected laboratory parameter in most institutions. However, vitamin D and FGF-23 levels are not typically day-to-day clinical measurements. Therefore, serum calcium provides a more convenient parameter as a clinical predictor of kidney disease progression.

Several potential mechanisms could explain our study observations. First, hypocalcemia, hyperparathyroidism, and vitamin D deficiency could be associated with increased circulating FGF-23 levels and renal functional deterioration. Studies have shown an inverse association between renal function and FGF-23 levels in CKD [20, 22]. Isakova et al. evaluated a large prospective cohort of patients with CKD stages 2–4, and identified that elevated FGF-23 is an independent risk factor for end-stage renal disease (ESRD) [23]. Nakano et al. further reported that increased FGF-23 was significantly associated with higher risk of ESRD, irrespective of CKD stage, and that serum calcium was associated with renal events after adjusting for FGF-23 [24].

In an advanced CKD population, suboptimal levels of 1,25(OH)_2_D_3_ are frequently observed. Experimental and epidemiologic data have emphasized the contribution of a deficiency in vitamin D toward impaired kidney function [25, 26]. Low plasma 1,25(OH)_2_)D increases the likelihood of requiring long-term dialysis in patients with advanced CKD [8]. De Boer et al. observed a low serum 25-hydroxyvitamin D concentration (25(OH)D) and rapid reduction in GFR in older adults with normal baseline kidney function [27]. Therefore, hypocalcemia might indicate the severity of vitamin D deficiency and emphasize its pathophysiological role in renal functional decline.

During early CKD, iPTH levels are typically elevated and might contribute to various complications [28]. Hypocalcemia in CKD can be associated with secondary hyperparathyroidism (SHPT). Previous studies have shown that higher mortality was associated with SHPT in maintenance hemodialysis patients [29, 30] and in a cohort of men with CKD stages 3–5 [31]. The association between SHPT and renal function progression is not well-characterized. In a nephrectomized animal model, iPTH accelerated CKD progression in rats with a high-protein diet [32]. In a small cohort of diabetic pre-dialysis patients, SHPT was associated with a higher rate of CKD progression [33]. In our cohort, low serum calcium remains significantly associated with RRT and rapid renal function progression after adjustment for covariates (Table 3).

Calcium ion is responsible for the nerve and synaptic excitation as well as cardiac myocytes contraction [34]. The effect of hypocalcemia on myocardial cells is unclear, but we know that the excitation-contraction coupling of the heart depends on the flow of ionized calcium [35]. In the complicated metabolic environment of CKD patients, the direct effect of serum calcium on cardiac performance remained controversial. In CKD patients, Gromadzinski L et al. have shown that hypocalcemia is related to left ventricular diastolic dysfunction [34]. In our study, low serum calcium was associated with lower MAP and higher percentage of LVMI (Table 1). Therefore, this association might have contributed to rapid renal function progression and the requirement for RRT.

Investigators have proposed that calcium overload plays a direct role in the pathogenesis of acute kidney injury by altering cellular function [11]. In a population of hemodialysis patients, Tentori et al. observed that serum calcium >10 mg/dL was associated with the highest risk of mortality [36]. However, in our study, high serum calcium (>9.8 mg/dL) did not show an association with lower eGFR at baseline or with poor renal outcomes at follow-up [2, 37]. These results could be attributed to fewer hypercalcemic patients, and low usage of calcium-containing phosphate binders, in our cohort.

Numerous epidemiological studies have shown a correlation between Ca × P and cardiovascular outcome and mortality [3, 38, 39]. The role of Ca × P in CKD progression remains controversial. Schwarz et al. showed that higher Ca × P was associated with higher risk of progressive CKD [2]. However, the role of Ca × P as an independent risk factor for morbidity and mortality is questionable because serum phosphorus, which is well-known as a novel risk factor for kidney disease progression and mortality in CKD, could have predominantly contributed to the variability in the parameter [4, 40]. Tentori F et al. in The Dialysis Outcomes and Practice Patterns Study (DOPPS) demonstrated that the combination of high calcium and high phosphorus accounted for the greatest risk of mortality in dialysis patients [36]. In Our study, patients with high serum phosphorus (>4.2 mg/dL) in combination with low serum calcium (<9.1 mg/dL) were associated with the highest risk of kidney disease progression. Our results suggested that the combination of serum phosphorus and calcium may provide a more accurate clinical marker than serum phosphorus or calcium alone does. Whether calcium should be regarded as a risk factor in the risk model would need further study to verify.

Our study has several limitations. First, it is a cohort study and not a randomized controlled trial. Therefore, our results showed only the association between serum calcium and renal outcome. In our cohort, patient with low serum calcium have low GFR and more rapid decline in renal function. However, there are many precipitated comorbidities which contribute to poor GFR. Further investigation is required to identify the mechanism(s) underlying serum calcium and kidney disease progression. Second, our study cohort was an advanced CKD population. Therefore, our study results might not be applicable in earlier stage CKD patients. Third, we did not measure vitamin D and FGF-23 levels. In our patients, the use of vitamin D and calcium-containing phosphate binders was relatively low. Therefore, we were unable to identify the effects of these compounds. Fourth, cardiac echography examination is only applicable to patients who entered CKD stage 5. However, previous studies that evaluated vitamin D and FGF-23 have shown the prognostic value of calcium in renal disease. Fifth, ionized calcium was not measured in our study. Further larger investigations should be conducted to confirm these results.

## Conclusion

In conclusion, our study results show that low serum calcium is associated with increased risk of requiring RRT and rapid renal function progression. The integration of serum calcium and phosphorus, but not calcium-phosphorus product should be considered in a predictive model of renal outcome.

## Electronic supplementary material

Additional file 1: Table S1: **Odds ratios for LVH (LVMI > 140) according to quartiles of serum calcium level.** Adjusts the variables including age, gender, eGFR, log(UPCR), diabetes mellitus, cardio vascular disease, HbA1c, mean BP, hemoglobin, albumin, log(cholesterol), ln(CRP), body mass index, phosphate binder and PTH. Abbreviations: LVH, Left ventricular hypertrophy; LVMI, left ventricular mass index; eGFR, estimated glomerular filtration rate; UPCR, Urine protein to creatinine ratio; HbA1c, glycosylated hemoglobin; BP, blood pressure; PTH, Parathyroid hormone. (DOC 40 KB)
