# Supplementary material for: Low serum calcium is associated with poor renal outcomes in chronic kidney disease stages 3–4 patients
Source: BMC Nephrol. 2014 Nov 21;15:183. doi: 10.1186/1471-2369-15-183 (PMC4255427; doi:10.1186/1471-2369-15-183)
Supplement: Supplementary file 1 — Additional file 1: Table S1: Odds ratios for LVH (LVMI > 140) according to quartiles of serum calcium level. Adjusts the variables including age, gender, eGFR, log(UPCR), diabetes mellitus, cardio vascular disease, HbA1c, mean BP, hemoglobin, albumin, log(cholesterol), ln(CRP), body mass index, phosphate binder and PTH. Abbreviations: LVH, Left ventricular hypertrophy; LVMI, left ventricular mass index; eGFR, estimated glomerular filtration rate; UPCR, Urine protein to creatinine ratio; HbA1c, glycosylated hemoglobin; BP, blood pressure; PTH, Parathyroid hormone. (DOC 40 KB) [file 12882_2014_872_MOESM1_ESM.doc]

**Additional file 1: Table S1 Odds ratios for LVH (LVMI>140) according to quartiles of serum calcium level**

|  | **Quartile of serum calcium level (mg/dL)** | | | |
| --- | --- | --- | --- | --- |
|  | **Q_1_** | **Q_2_** | **Q_3_** | **Q_4_** |
|  | **(< 9.0)** | **(9.0 - 9.4)** | **(9.4 - 9.8)** | **(> 9.8)** |
| Unadjusted OR (95% CI) | 1.58 (0.92-2.78) | 1.26 (0.70-2.15) | 1 (reference) | 1.29 (0.75-2.22) |
| Adjusted OR (95% CI) | 1.64 (0.98-2.95) | 1.28 (0.90-2.75) | 1 (reference) | 1.07 (0.55-2.12) |

Adjusts the variables including age, gender, eGFR, log(UPCR), diabetes mellitus, cardio vascular disease, HbA1c, mean BP, hemoglobin, albumin, log(cholesterol), ln(CRP), body mass index, phosphate binder and PTH.

LVH, left ventricular hypertrophy; LVMI, left ventricular mass index; eGFR, estimated glomerular filtration rate; UPCR, Urine protein to creatinine ratio; HbA1c, glycosylated hemoglobin; BP, blood pressure; PTH, parathyroid hormone.
